# Biological aging across the metabolic dysfunction–associated steatotic liver disease spectrum: A systematic review

**DOI:** 10.1016/j.iliver.2026.100222

**Published:** 2026-02-04

**Authors:** Chukwuemeka E. Ogbu, Stella C. Ogbu, Chidera P. Ogbu, Chinazor Umerah

**Affiliations:** a Internal Medicine Residency Program, Cape Fear Valley Health, Fayetteville, NC 28304, USA; b Department of Biochemistry and Molecular Biology, Medical College of Augusta, Augusta, GA 30912, USA

**Keywords:** Biological age, Klemera-doubal method, Epigenetic clock, Phenotypic age, MASLD, NAFLD, NASH, Fibrosis, Mortality, Geroscience, Systematic review

## Abstract

**Background and aims:**

Metabolic dysfunction–associated steatotic liver disease (MASLD) is a growing global health burden. Geroscience posits that accelerated biological aging is a key driver of chronic disease. We systematically reviewed the evidence to define the role of biological aging (BA) across the MASLD disease spectrum.

**Methods:**

A systematic literature search of PubMed/MEDLINE and Embase was conducted from inception through August 2025 for observational studies in adults assessing validated BA measures (phenotypic age, Klemera–Doubal method, Homeostatic Dysregulation, epigenetic clocks, telomere length) and MASLD outcomes. A narrative synthesis was performed following SWiM guidelines because of substantial methodological heterogeneity.

**Results:**

Nineteen studies were included. BA was operationalized using clinical composite clocks (KDM-BA, PhenoAge, Homeostatic Dysregulation), epigenetic clocks, leukocyte telomere length, homeostatic dysregulation indices, and one machine-learning BA metric. Accelerated BA generally referred to BA higher than expected for chronological age or shorter telomere length. Across large cross-sectional studies, individuals with accelerated BA had higher odds of MASLD/nonalcoholic fatty liver disease (NAFLD), and in prospective cohorts, higher BA at baseline predicted increased hazards of incident NAFLD after multivariable adjustment, suggesting BA functions as an upstream integrator of risk rather than only a consequence of liver disease. Accelerated BA was also associated with greater fibrosis burden and with higher all-cause mortality among people with MASLD. Several studies indicated that BA mediates part of the effect of environmental toxicants on MASLD and acts as an effect modifier, with higher risk observed when accelerated BA co-occurs with unfavorable genetic profiles or environmental exposures. Mendelian randomization analyses supported a potential causal role of cellular aging in liver fibrogenesis.

**Conclusion:**

Across 19 observational studies, accelerated biological aging (assessed using clinical composite indices, epigenetic clocks, and telomere length) was consistently associated with higher MASLD/NAFLD risk, greater fibrosis severity, and higher mortality. These findings support BA as potentially a clinically relevant risk integrator in MASLD spectrum diseases. However, heterogeneity in BA measures and disease definitions limits comparability and highlight the need for harmonized BA measures in longitudinal studies.

## 1 Introduction

Metabolic dysfunction–associated steatotic liver disease (MASLD), the updated term in place of non-alcoholic fatty liver disease (NAFLD), situates hepatic steatosis within its cardiometabolic framework and establishes standardized case definitions for both clinical research and practice.^1^ MASLD represents a significant portion of chronic liver disease globally. Currently, 38% of adults and between 7% and 14% of children and adolescents have MASLD.^2^ In the United States, the prevalence of MASLD is estimated to increase from 33.7% in 2020 to 41.4% by 2050, potentially affecting 122 million adults.^3^ This growth is expected to coincide with increases in metabolic dysfunction–associated steatohepatitis (MASH), advanced fibrosis, and related complications.^3^ While MASLD does not consistently progress to advanced liver disease, it is currently the leading reason for liver transplantation among women and individuals with hepatocellular carcinoma (HCC) in the United States.^2^ Lifestyle changes remain central to treatment, but new therapies are emerging. In March 2024, the FDA approved resmetirom for adults with noncirrhotic MASH and F2–F3 fibrosis, underscoring the considerable clinical impact and the growing need for better risk assessment beyond conventional factors.^4^^,^^5^

Aging is recognized as the primary risk factor for chronic diseases.^6^ However, chronological age does not fully account for variations in functional decline among individuals. The concept of biological age (BA) aims to measure organismal aging by using biochemical, molecular and epigenetic biomarkers that more accurately reflect morbidity and mortality risks than the number of years lived.^7^ Foundational measures of biological aging include biomarker-based algorithms such as the Klemera-Doubal method (KDM) and Phenotypic Age (PhenoAge) which are calibrated against mortality risk and quantify physiological decline.^8^^,^^9^ At the molecular level, epigenetic clocks such as Horvath DNAm age, DNAm PhenoAge, GrimAge, DunedinPACE, estimate age or pace-of-aging from DNA methylation patterns and are strongly associated with morbidity and mortality across populations.^10^, ^11^, ^12^ Telomere length also provides a related cellular marker of replicative aging.^13^ Together, these tools provide complementary windows into multisystem decline that characterize metabolic liver disease.

The accumulation of senescent cells is a key mechanism through which Biological Aging (BA) drives hepatic steatosis and fibrogenesis.^14^ Senescent cells impair metabolic function, promoting lipid accumulation, and secrete inflammatory and profibrotic factors known as the senescence-associated secretory phenotype (SASP), that propagate tissue injury and remodeling.^15^^,^^16^ Experimental studies show that increasing senescent-cell burden induces hepatic fat accumulation, whereas genetic or pharmacologic clearance of senescent cells mitigates steatosis and fibrotic remodeling.^17^^,^^18^ Consistent with this observation, obesity appears to accelerate epigenetic aging in human liver tissue and associates metabolic stress with aging trajectories.^19^

Epidemiologic studies that link validated measures of BA to MASLD are emerging but heterogeneous. In a nationally representative U.S. sample, advanced liver fibrosis but not MALSD, was associated with accelerated BA using blood biomarker BA algorithms, suggesting that aging signatures intensify with disease severity.^20^ In the UK Biobank, longer leukocyte telomere length was associated with lower incident NAFLD risk, consistent with a younger cellular age phenotype.^21^ Studies that measured BA as epigenetic clocks report age acceleration in biopsy-proven NAFLD/Non-Alcoholic Steatohepatitis (NASH) and correlate methylation signatures to hepatic outcomes.^22^^,^^23^ Another study showed that BA acceleration derived from clinical biomarkers predicts incident NAFLD independent of polygenic risk which highlights another partially distinct pathway from inherited susceptibility.^24^

Although significant progress has been made in the science of BA and MASLD has long been recognized as an age-related condition,^25^ observational evidence remains fragmented due to variations in study designs, populations, and measurement approaches. The literature reveals different methods for assessing BA, such as blood composite biomarkers, epigenetic clocks, and measurements of telomere length, as well as outcomes that range from simple steatosis to advanced fibrosis and mortality.^26^ Therefore, a systematic synthesis of this evidence is appropriate at this time for three main reasons. Firstly, validated BA measures have the potential to enhance risk stratification beyond traditional factors and standard noninvasive tests, enabling more targeted screening, surveillance, and prevention. Secondly, because BA quantifies modifiable biological processes like senescence and inflammaging, it can directly inform therapeutic strategies from weight loss and GLP-1 agonists to future senolytics and provide important biomarkers for tracking their efficacy. Third, there has not been a systematic review of current evidence till date that evaluates these associations.

To address this need, we conducted a systematic review to assess observational studies examining the association between BA and MASLD, as well as related severity, incidence, and clinical outcomes. This review synthesizes current evidence and establishes a foundation for future investigations aimed at reducing MASLD burden from the standpoint of biological aging.

## 2 Methods

The protocol for this systematic review was developed in accordance with the Preferred Reporting Items for Systematic Reviews and Meta-Analyses (PRISMA) guidelines.^27^ This review was not registered in PROSPERO or another registry. The protocol was developed a priori by the study team and the review was conducted according to PRISMA guidance. The aim was to synthesize evidence on the relationship between biological aging and the MASLD spectrum, including MASLD/NAFLD/NASH/fatty liver disease. We included observational studies that evaluated biological aging and MASLD in adult participants.

### 2.1 Eligibility criteria

We included studies that met all of the following criteria: (a) observational human design—prospective or retrospective cohort, case-control, or analytical cross-sectional—and randomized clinical trials if they measured biological aging as a key variable (e.g., exposure, mediator, effect modifier, or outcome); (b) participants aged ≥18 years; (c) a validated biological aging measure, including clinical composite ages (e.g., Klemera–Doubal biological age, Phenotypic Age or equivalents), epigenetic clocks (e.g., Horvath, DNAm PhenoAge, GrimAge, DunedinPACE or equivalents), or leukocyte telomere length analyzed as a primary exposure, mediator (with indirect and direct effects quantified), effect modifier (with a formal interaction), or outcome; and (d) MASLD/NAFLD/fatty liver disease/MASH/NASH defined by imaging or elastography (ultrasound, MRI-PDFF, VCTE/MRE with standard quality metrics), biopsy, registry/ICD codes or clinician diagnosis with documented coding validity, or validated indices (e.g., FLI/US-FLI) with appropriate exclusions for secondary causes and excess alcohol. We restricted inclusion to English-language studies in adults, with searches run through August 2025. Full inclusion and exclusion criteria are summarized in Table 1.

**Table 1:** The inclusion and exclusion criteria for study selection.

Category	Inclusion criteria	Exclusion criteria
Study design	Observational human studies: prospective or retrospective cohort, case-control, analytical cross-sectional. Randomized clinical trials will be considered if they measure biological aging as an exposure.	Case reports/series, narrative reviews, editorials, animal or in vitro studies, conference abstracts, and pre-prints.
How BA is quantified	Studies quantifying biological aging with validated measures: clinical composite ages (e.g., Klemera-Doubal, Phenotypic Age), epigenetic clocks (e.g., Horvath, DNAm PhenoAge, GrimAge, DunedinPACE, or other validated clocks), leukocyte telomere length; or acceleration metrics.	No biological aging metric; single biomarkers only (e.g., ALT, CRP) without a validated composite algorithm; chronological age alone; frailty or comorbidity indices used as surrogates.
Role BA	Any of the following: (a) primary exposure predicting MASLD outcomes; (b) mediator in a specified pathway; (c) effect modifier of another exposure; (d) an outcome itself.	BA used only as an adjustment covariate; descriptive correlations without formal modeling.
Comparison	Lower vs. higher biological age with a reported adjusted association (OR/HR/RR) and 95% CI (or data to derive it), or BA analyzed as a continuous variable.	Unadjusted descriptive reports without effect estimates.
Spectrum of MASLD captured	MASLD/NAFLD presence, incidence, or severity (advanced fibrosis, cirrhosis, HCC, liver-related mortality).	Alcoholic liver disease only; secondary steatosis without a separable MASLD/NAFLD subgroup; non-hepatic outcomes.
MASLD/NAFLD definition	Imaging (e.g., ultrasound, MRI-PDFF; VCTE CAP/LSM), validated indices (e.g., FLI) with appropriate exclusions, or registry/ICD codes in studies with demonstrated coding validity.	No explicit definition; liver enzymes alone; self-report without validation.
Language of publication	English.	All other languages.
Participants	Human adults (≥18 years).	Non-human subjects; children/adolescents (<18 years).
Age of participants	Adults ≥18 years (including older adults)	Children/adolescents <18 years
Date of publication	No lower limit, through the end of August 2025.	—

Footnote: BA, biological aging; MASLD, metabolic dysfunction–associated steatotic liver disease; NAFLD, nonalcoholic fatty liver disease; HCC, hepatocellular carcinoma; MRI-PDFF, magnetic resonance imaging–proton density fat fraction; VCTE, vibration-controlled transient elastography; CAP, controlled attenuation parameter; LSM, liver stiffness measurement; FLI, Fatty Liver Index; ICD, International Classification of Diseases; DNAm, DNA methylation; OR, odds ratio; HR, hazard ratio; RR, risk ratio (relative risk); CI, confidence interval; ALT, alanine aminotransferase; CRP, C-reactive protein.

### 2.2 Search strategy

A systematic literature search was performed in PubMed/MEDLINE and Embase from inception through August 31, 2025. The search strategy was designed to capture all relevant terms for NAFLD/MASLD and biological aging. The search combined MeSH and non-MeSH terms and was developed by investigators with content expertise. The NAFLD/MASLD component included the following terms: (“non-alcoholic fatty liver disease” [MeSH Terms] OR NAFLD [Title/Abstract] OR “non-alcoholic steatohepatitis” [Title/Abstract] OR NASH [Title/Abstract] OR MASLD [Title/Abstract] OR “metabolic dysfunction associated steatotic liver disease” [Title/Abstract] OR “hepatic steatosis” [MeSH Terms]). The biological aging component included: (“biological aging” [Title/Abstract] OR “biological age” [Title/Abstract] OR “phenotypic age” [Title/Abstract] OR PhenoAge [Title/Abstract] OR “Klemera-Doubal” [Title/Abstract] OR “telomere length” [MeSH Terms] OR “telomere shortening” [Title/Abstract] OR “epigenetic clock” [Title/Abstract] OR “DNA methylation age” [Title/Abstract] OR “age acceleration” [Title/Abstract] OR “allostatic load” [Title/Abstract] OR “homeostatic dysregulation” [Title/Abstract]). We also manually screened the reference lists of all included studies and relevant review articles to identify additional eligible publications.

### 2.3 Study selection

**Fig. 1:**
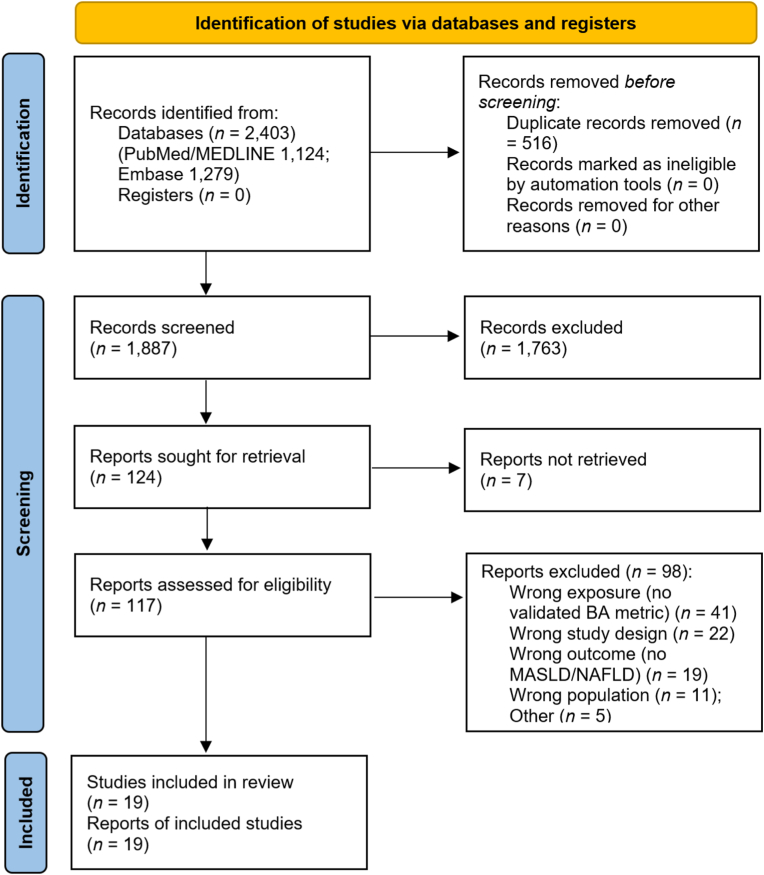
**PRISMA 2020 flow diagram of study identification, screening, eligibility assessment, and inclusion**. Records were identified from PubMed/MEDLINE (*n* ​= ​1124) and Embase (*n* ​= ​1279). After removal of duplicates (*n* ​= ​516), 1887 records were screened, and 1763 were excluded at title/abstract screening. Full texts were sought for 124 reports; 7 were not retrieved. A total of 117 full-text reports were assessed for eligibility, and 98 were excluded (wrong exposure/no validated biological aging metric [*n* ​= ​41], wrong study design [*n* ​= ​22], wrong outcome/no MASLD/NAFLD [*n* ​= ​19], wrong population [*n* ​= ​11], other [*n* ​= ​5]). Nineteen studies were included in the final review. **Abbreviations:** MASLD, metabolic dysfunction–associated steatotic liver disease; NAFLD, nonalcoholic fatty liver disease; BA, biological aging.

Search results were imported into EndNote X9 for deduplication. Two independent reviewers (C.E.O. and S.C.O.) screened titles and abstracts against the eligibility criteria. Full texts of potentially relevant articles were then retrieved and assessed in detail. Any disagreements at either stage were resolved through discussion and consensus. The study selection process is summarized in a flow diagram (Fig. 1).

### 2.4 Data extraction and quality assessment

Two reviewers (C.E.O. and S.C.O.) independently screened titles and abstracts, reviewed full texts, and selected studies. Disagreements were resolved by discussion with a senior reviewer. From each eligible paper, we abstracted: first author and year; country and source sample; sample size and population characteristics; study design and follow-up window (for cohort studies); definition of MASLD/NAFLD and thresholds used; BA measure, including how algorithm inputs were reported, how aging was operationalized, and the role of BA in the model (exposure, mediator, effect modifier, or outcome). We also recorded statistical methods, covariates adjusted for in the analysis, and adjusted effect estimates (odds ratios, hazard ratios, or risk ratios) with 95% confidence intervals.

Risk of bias was assessed independently by the same two reviewers. For cohort and case-control studies, we used the Newcastle-Ottawa Scale (NOS), which awards a maximum of 9 points across domains of selection, comparability, and outcome/exposure.^28^ Studies scoring ≥7 points were considered high quality. For analytical cross-sectional studies, we used an adapted NOS version, cross-checked against the JBI critical appraisal checklist to evaluate representativeness, measurement validity, confounding, and outcome assessment.^29^ Discrepancies in scoring were resolved by consensus with the senior reviewer.

### 2.5 Synthesis without meta-analysis

Due to significant methodological heterogeneity in exposure and outcome measurements across studies including the use of different biological aging algorithms and various methods for defining MASLD and fibrosis, a meta-analysis was deemed inappropriate. Therefore, a narrative synthesis was conducted following the Synthesis Without Meta-Analysis (SWiM) guidelines.^30^ Findings were structured thematically by outcome (prevalence, incidence, fibrosis, mortality) and the role of biological aging (exposure, mediator, effect modifier and outcome). Given expected heterogeneity across biological aging measures and steatotic liver disease definitions (NAFLD/MAFLD/MASLD, imaging vs indices vs ICD codes), we emphasized within-study adjustment, direction and consistency of associations, and we discuss potential selection and measurement biases related to dominant data sources. Table 2 shows the Newcastle-Ottawa Scale Quality Assessment of included studies. Table 3 shows the synthesis table of included studies.

**Table 2:** Newcastle–ottawa scale quality assessment of included studies examining biological aging and MASLD.

Study (author, year [ref])	NOS model/design	Selection (0–4)	Comparability (0–2)	Outcome/Exposure (0–3)	Total (0–9)	Overall risk
Zhao et al., 2025^31^	Cross-sectional	3	2	2	7	Moderate
Tong et al., 2024^20^	Cross-sectional	3	2	2	7	Moderate
Liu et al., 2025^32^	Cross-sectional	3	2	2	7	Moderate
Zhao & Wang et al., 2025^24^	Cohort	4	2	2	8	Low
Deng et al., 2025^33^ ∗	Cohort (NHANES)	3	2	2	7	Moderate
Guo et al., 2025^34^	Cohort	4	2	2	8	Low
Zhang & Ding et al., 2025^35^	Cross-sectional	3	2	2	7	Moderate
Loomba et al., 2018^22^	Case–control	3	2	2	7	Moderate
Xia et al., 2024^23^	Cohort	3	2	2	7	Moderate
Tang et al., 2023^21^	Cohort	4	2	2	8	Low
Wu et al., 2025^36^	Cross-sectional	3	2	1	6	Moderate
Zhang & Hai et al., 2025^37^	Cohort	4	2	2	8	Low
Wang & Liu et al., 2024^38^ ∗	Cohort (NHANES III)	4	2	2	8	Low
Kim et al., 2024^39^ ∗	Cohort (NHANES)	3	2	2	7	Moderate
Gawrieh et al., 2025^40^	Cross-sectional	3	2	1	6	Moderate
Ping et al., 2017^41^	Cohort	2	1	2	5	High
Peng et al., 2025^42^	Cross-sectional	3	2	1	6	Moderate
Van Dijck et al., 2025^43^	Cross-sectional	2	1	2	5	High
Yang et al., 2025^44^	Cross-sectional	3	2	1	6	Moderate

Footnote. NOS = Newcastle–Ottawa Scale. Quality was assessed separately for cohort, case–control, and cross-sectional analytic designs using the NOS, with domain scores for Selection (0–4 stars), Comparability (0–2 stars), and Outcome/Exposure (0–3 stars), yielding a maximum of 9 points. For cross-sectional studies, an accepted NOS adaptation was applied, with Selection items mapped to representativeness and ascertainment of exposure/outcome, Comparability to control for key confounders, and Outcome/Exposure to measurement quality and statistical handling. Overall risk of bias was categorized as low (8–9 points), moderate (6–7 points), or high (≤5 points). ∗ Three NHANES analyses: Deng et al.(2025),^33^ Wang & Liu et al. (2024),^38^ and Kim et al. (2024)^39^ were treated as cohort studies for NOS scoring because baseline survey data were linked to prospective mortality follow-up.

**Table 3:** Table of selected studies.

Author, year [ref]	Data source/country	Design & sample	Biological aging measure(s)	Liver related definition & measures	Role of BA in analysis	Key adjusted findings∗
Zhao et al., 2025^31^	NHANES 2017–2020 (U.S.) and 2-sample MR	Cross-sectional sample of adults ≥18 year. MASLD, *n* ​= ​2071	1. Phenotypic Age 2. PhenoAgeAccel (PhenoAge ​− ​chronological age)	MASLD defined by CAP ≥248 ​dB/m or FLI ≥30 ​+ ​≥ 1 cardiometabolic factor Fibrosis by LSM ≥8.2 ​kPa	BA as primary exposure Fibrosis as outcome	Each +1 year PhenoAgeAccel is associated with higher odds of significant fibrosis (OR ​= ​1.01 per year; OR ​= ​1.08 for PhenoAgeAccel >0 vs ​≤ ​0) MR suggested longer telomere length causally related to lower fibrosis/cirrhosis risk.
Tong et al., 2024^20^	NHANES 2017–2018 (U.S.)	Cross-sectional sample of adults 20–85 year (*n* ​= ​3974)	KDM Biological Age and PhenoAge, expressed as “advance” (BA ​− ​chronological age)	Steatosis (CAP ≥288 ​dB/m) Advanced fibrosis (LSM ≥9.7 ​kPa) MASLD defined as steatosis + ​≥ ​1 cardiometabolic factor	BA as outcome AF/MASLD as exposures	Advanced fibrosis, but not MASLD itself, associated with higher BA AF vs non-AF associated with +1.5 years KDM_advance and about +1.0 year PhenoAge_advance after adjustment.
Liu et al., 2025^32^	NHANES 2005–2016 (U.S.)	Cross-sectional adults (*n* ​= ​2786; NAFLD 1116)	KDM-BA, Phenotypic Age, Homeostatic Dysregulation, Allostatic Load	NAFLD by FLI ≥60	BA as primary exposure NAFLD prevalence as outcome	With per 1-SD higher BA, odds of NAFLD increased (PhenoAge OR 1.76; AL OR 1.19; HD OR 1.21), while KDM-BA showed a weaker, non-significant association.
Zhao & Wang et al., 2025^24^	UK Biobank (U.K.) and Dongfeng–Tongji Cohort (China)	Two prospective cohorts: UKB *n* ​= ​329,040, follow-up ​= ​12.45 years DTC *n* ​= ​6,783, follow-up ​= ​10 years	KDMAge and PhenoAge BA acceleration (residuals from BA ​to ​chronological age)	Incident NAFLD/NASH via ICD codes (UKB) or ultrasound-defined NAFLD (DTC) with exclusion of secondary causes	BA as primary exposure Incident NAFLD as outcome PRS as effect modifier	Accelerated BA predicted higher incident NAFLD: in UKB, each +1 year BA acceleration increased risk by 6%, and accelerated” vs “non-aging” BA raised risk by about 35 to 70% In DTC, accelerated BA was also associated with higher NAFLD odds (OR ​= ​1.2) PRS not a clear effect modifier
Deng et al., 2025^33^	Hospital cohorts in China and NHANES 1999–2018 (U.S.)	Cross-sectional BA model development in Chinese hospital cohorts (*n* ​= ​39,774 adults) with a separate ultrasound-defined NAFLD cohort (*n* ​= ​1663), plus external validation and NAFLD mortality follow-up in NHANES 1999–2018 (NAFLD *n* ​= ​1445; mortality subcohort *n* ​= ​1439).	Novel ML-derived biological age (“NC-BA”) BA acceleration defined as standardized age deviation, moderately correlated with KDM, PhenoAge, and HD.	Ultrasound-defined NAFLD in clinical cohort CAP-defined NAFLD in NHANES with standard exclusions	BA as exposure for NAFLD status and mortality	NAFLD patients had higher BA acceleration (SAD) than non-NAFLD in both Chinese and NHANES cohorts. In NHANES NAFLD, higher SAD strongly predicted all-cause mortality (high vs low SAD HR ​= ​6 overall and 14 in men; per-unit SAD HR ​= ​1.7 overall).
Guo et al., 2025^34^	UK Biobank (U.K.)	Prospective cohort (*n* ​= ​277, 057; NAFLD cases 2921; cirrhosis 2113; follow-up about 12 years)	KDMAgeAccel and PhenoAgeAccel (BA ​− ​chronological age)	Incident NAFLD and cirrhosis via ICD codes	BA as main exposure NAFLD & cirrhosis as outcomes PRS as modifier	Per SD increase in BA acceleration, HRs for incident NAFLD and cirrhosis ranged from 1.17 to 1.37 Highest BA quartile had 1.5–1.7-fold higher NAFLD risk and 1.4–1.8-fold higher cirrhosis risk vs lowest Risk highest in those with both high BA and high genetic risk.
Zhang & Ding et al., 2025^35^	NHANES 2017–2018 (U.S.)	Cross-sectional adults (*n* ​= ​2421; NAFLD 843)	KDM Biological Age	NAFLD by US Fatty Liver Index (FLI ≥60) and noninvasive fibrosis scores	BA as exposure NAFLD and fibrosis indices as outcomes HEI-2015 as mediator	Higher BA associated with higher NAFLD odds in a dose–response pattern (Q4 vs Q1 OR ​= ​2.5) Better diet quality attenuated the BA–NAFLD link by a small but significant proportion (6%).
Loomba et al., 2018^22^	Single-center biopsy cohort ​+ ​external controls	Case–control Biopsy-proven NASH with F2–F3 fibrosis (*n* ​= ​44) vs multiple control groups (*n* ​= ​718 total)	Peripheral-blood DNA methylation Horvath epigenetic clock Age acceleration (residuals)	NASH and fibrosis stage from liver biopsy Collagen content and ELF as fibrosis markers	BA as exposure NASH case status and fibrosis severity as outcomes	DNAm age acceleration was higher in NASH cases than in controls and correlated with histologic fibrosis measures Positive correlations with collagen content and ELF scores.
Xia et al., 2024^23^	Shanghai Changfeng community cohorts (China)	Prospective cohorts (*n* ​= ​95 with incident T2DM and *n* ​= ​356 general sample)	Blood DNAm Horvath clock DNAm age acceleration (DNAm age ​− ​chronological age)	Fatty liver by quantitative ultrasound algorithm (liver fat content) NAFLD/MAFLD with standard exclusions	BA as exposure Incident NAFLD/MAFLD and change in liver fat as outcomes	Higher epigenetic age acceleration associated with substantially higher odds of incident NAFLD/MAFLD (ORs between 4 and 6 for highest vs lowest tertile) and with greater longitudinal increases in liver fat content.
Tang et al., 2023^21^	UK Biobank (U.K.)	Prospective cohort (*n* ​= ​467,848; NAFLD cases 4809; median follow-up ​= ​12.83 years)	Leukocyte telomere length (LTL), z-standardized	Incident NAFLD and NASH by ICD MRI-PDFF to define NAFLD in imaging subset	BA proxy (LTL) as exposure NAFLD as outcome Mediator between age/lifestyle/air pollution and NAFLD	Longer leukocyte telomere length was modestly protective for NAFLD (HR ​= ​0.93 per IQR; 13% lower risk in the longest vs shortest quartile), with similar inverse associations for MRI-PDFF NAFLD subset and no clear effect for NASH. Telomere length mediated 15% of the age–NAFLD association, and individuals with both short telomeres and high NAFLD polygenic risk had the highest NAFLD risk, consistent with modest additive interaction.
Wu et al., 2025^36^	NHANES 1999–2018 (U.S.)	Cross-sectional adults ≥20 years (*n* ​= ​5205; MASLD *n* ​= ​2307)	KDM Biological Age and Phenotypic Age	MASLD by US-FLI/FLI-based fatty liver with standard exclusions	BA as mediator between environmental exposures and MASLD	Higher urinary metal mixtures were associated with higher MASLD odds BA (both KDM-BA and PhenoAge) strongly predicted MASLD (Q4 vs Q1 OR ​= ​2.8–2.9) and mediated 20–26% of the metals–MASLD association, with even higher mediation proportions for some individual metals.
Zhang & Hai et al., 2025^37^	UK Biobank (U.K.)	Prospective cohort (*n* ​= ​296,917; incident NAFLD 5109; follow-up ​= ​11.9 years)	KDM-BA and PhenoAge; BA acceleration	NAFLD/NASH by ICD codes	BA as a primary exposure Joint effects and interaction of BA with PM_2.5_ evaluated.	Both higher BA and higher PM_2.5_ independently predicted incident NAFLD (BA HRs ​= ​1.4–1.5 per SD; PM_2.5_ HR ​= ​1.07 per SD). Participants with both high BA and high PM_2.5_ had the greatest NAFLD risk with positive additive interaction.
Wang & Liu et al., 2024^38^	NHANES III NAFLD cohort (U.S.)	Prospective NAFLD cohort (*n* ​= ​2199; median follow-up 16 years)	KDM age, PhenoAge, and Homeostatic Dysregulation Acceleration metrics	NAFLD by standardized ultrasound criteria with exclusions for other liver diseases and excess alcohol	BA as exposure All-cause and cause-specific mortality as outcomes	Among people with NAFLD, higher BA and HD predicted higher all-cause mortality PhenoAge acceleration showed particularly strong associations, and HD was strongly associated with diabetes-related mortality.
Kim et al., 2024^39^	NHANES 1999–2002 (U ​S)	Prospective MASLD cohort (baseline NHANES survey; *n* ​= ​3618 adults) with mortality follow-up through 2019 (median 18.4 years).	Leukocyte telomere length (quartiles)	SLD by HSI ≥36 MASLD ​= ​SLD ​+ ​≥1 cardiometabolic criterion	LTL as exposure All-cause and cause-specific mortality as outcomes	Shorter LTL (higher quartile number) was associated with higher all-cause mortality in MASLD (Q4 vs Q1 HR ​≈ ​1.4), with stronger effects in older adults and men Trends for CVD/cancer mortality were positive but not significant
Gawrieh et al., 2025^40^	MESA (U.S.)	Cross-sectional CT–DNAm substudy (*n* ​= ​887; MASLD 139; PNPLA3 G-allele carriers 398)	Four epigenetic clocks (DNAmAge, Hannum, PhenoAge, GrimAge) Epigenetic age acceleration (EAA)	MASLD by CT liver attenuation plus metabolic criteria and exclusion of secondary causes	BA as exposure MASLD as outcome PNPLA3 genotype as modifier	Higher Hannum epigenetic age and its acceleration were associated with MASLD in minimally-adjusted models (ORs ​= ​1.04 per unit), with attenuation after full adjustment. EAA remained higher in MASLD vs controls and showed stronger associations among PNPLA3 G-allele carriers.
Ping et al., 2017^41^	Single-center T2DM clinic (China)	Prospective cohort (*n* ​= ​70; 6-year follow-up; 39 incident NAFLD)	Leukocyte telomere length measured at baseline and 6 years, measured as % shortening over time	Incident NAFLD by quantitative ultrasound hepatorenal ratio with exclusions	Telomere shortening as exposure Incident NAFLD as outcome	Greater telomere shortening over 6 years predicted higher odds of incident NAFLD (OR ​= ​1.5 per 1% shortening) independent of BMI, insulin, and blood pressure.
Peng et al., 2025^42^	NHANES 2017–2020 adults ≥50 ​y ​(U ​S.)	Cross-sectional (*n* ​= ​3212) with BMI trajectories	Phenotypic Age (Levine)	NAFLD by CAP ≥288 ​dB/m Advanced fibrosis by LSM 12–20 ​kPa Cirrhosis by LSM ≥20 ​kPa.	BA as mediator between BMI trajectories and MASLD outcomes	Rapidly ascending BMI trajectories were associated with higher odds of NAFLD and advanced fibrosis A portion of this association was mediated through higher Phenotypic Age and inflammatory markers (hs-CRP), with significant chain mediation.
Van Dijck et al., 2025^43^	Academic liver clinic, Antwerp (Belgium)	Cross-sectional biopsy-based DNAm cohort of 22 overweight/obese adults (no MASLD, MASL, MASH F0–F4) plus a separate expression cohort (*n* ​= ​118) with matched histology	Liver DNA methylation Epigenetic clocks (Horvath pan-tissue, LiverClock, HepClock, and Horvath IEAA) Epigenetic age acceleration (EAA)	Histologic MASLD spectrum (no MASLD, MASL, MASH F0–F4) by NASH CRN Steatosis, ballooning, and fibrosis grades as histologic outcomes	BA as exposure MASLD stage and histologic features as outcomes	Horvath EAA increased across MASLD stages and was positively correlated with steatosis, ballooning, and fibrosis after FDR correction LiverClock EAA related to MASLD stage only, while IEAA and HepClock EAA were not significant. CpGs associated with MASLD stage and with EAA showed substantial overlap, supporting liver epigenetic aging as a fibrosis-linked signal.
Yang et al., 2025^44^	NHANES 1999–2018 (U.S.)	Cross-sectional adults with MASLD (*n* ​= ​3045)	KDM Biological Age and Phenotypic Age	MASLD by HSI >36 or US-FLI ≥30 plus ≥1 cardiometabolic criterion Outcomes ​= ​insulin resistance, prediabetes, diabetes	BA both as exposure and effect modifier between phthalate exposure and glycemic outcomes	Higher BA and PA were associated with higher odds of insulin resistance, prediabetes, and diabetes among adults with MASLD, and participants with both high phthalate exposure and high PA had the greatest metabolic risk

**Abbreviations:** AF, advanced fibrosis; AL, allostatic load; BA, biological age; BA acceleration, biological age acceleration (BA ​− ​chronological age or residuals); CAP, controlled attenuation parameter; CI, confidence interval; CT, computed tomography; CVD, cardiovascular disease; DTC, Dongfeng–Tongji Cohort; DNAm, DNA methylation; EAA, epigenetic age acceleration; ELF, Enhanced Liver Fibrosis; FDR, false discovery rate; FLI, Fatty Liver Index; HD, homeostatic dysregulation; HEI-2015, Healthy Eating Index-2015; HR, hazard ratio; HSI, Hepatic Steatosis Index; ICD, International Classification of Diseases; IEAA, intrinsic epigenetic age acceleration; IQR, interquartile range; KDM-BA/KDMAge, Klemera–Doubal method biological age; LSM, liver stiffness measurement; LTL, leukocyte telomere length; MAFLD, metabolic dysfunction–associated fatty liver disease; MASLD, metabolic dysfunction–associated steatotic liver disease; MASH, metabolic dysfunction–associated steatohepatitis; MESA, Multi-Ethnic Study of Atherosclerosis; ML, machine learning; MRI-PDFF, magnetic resonance imaging–proton density fat fraction; MR, Mendelian randomization; NAFLD, nonalcoholic fatty liver disease; NASH, nonalcoholic steatohepatitis; NC-BA, novel machine-learning biological age algorithm; OR, odds ratio; PA, Phenotypic Age; PhenoAgeAccel, Phenotypic Age acceleration; PNPLA3, patatin-like phospholipase domain–containing protein 3; PRS, polygenic risk score; Q1/Q4, lowest/highest quartile; SAD, standardized age deviation (biological age acceleration metric); SD, standard deviation; SLD, steatotic liver disease; T2DM, type 2 diabetes mellitus; UKB, UK Biobank; US-FLI, United States Fatty Liver Index; PM_2.5_, fine particulate matter with aerodynamic diameter ≤2.5 ​μm; hs-CRP, high-sensitivity C-reactive protein.

## 3 Results

### 3.1 Study characteristics

Nineteen studies published between 2017 and 2025 met inclusion criteria 20, 21, 22, 23, 24^,^^31^, ^32^, ^33^, ^34^, ^35^, ^36^, ^37^, ^38^, ^39^, ^40^, ^41^, ^42^, ^43^, ^44^. They varied widely in design, population, and methods for assessing both liver disease and biological aging. Most were conducted in the United States and predominantly using National Health and Nutrition Examination Survey (NHANES) data either alone or with other datasets (12 studies). Four studies were conducted using the United Kingdom Biobank (UKB).^21^^,^^24^^,^^34^^,^^37^ Five studies used data from China, leveraging both large prospective cohorts and hospital-based data.^23^^,^^24^^,^^33^^,^^41^^,^^42^ One small, detailed epigenetic study was conducted on a Belgian biopsy cohort.^43^

The study designs were predominantly observational. Cross-sectional analyses were the most common (*n* ​= ​11^20^^,^^22^^,^^31^, ^32^, ^33^^,^^35^^,^^36^^,^^40^^,^^42^, ^43^, ^44^), followed by prospective cohort studies (*n* ​= ​7^21^^,^^23^^,^^24^^,^^34^^,^^37^, ^38^, ^39^). One study employed a case-control design.^22^ Sample sizes ranged from a detailed liver tissue epigenetics study (*n* ​= ​22)^43^ to large population-based cohorts (up to *n* ​= ​467,848 in UKB^21^).

Definitions of steatotic liver disease were heterogeneous. Frequently used noninvasive indices included the FLI, United States Fatty Liver Index (US-FLI), and Hepatic Steatosis Index (HSI). Several studies used ultrasound-based definitions, either semi-quantitative or quantified by hepatorenal ratio. Vibration-Controlled Transient Elastography (VCTE) with Controlled Attenuation Parameter (CAP) for steatosis and LSM for fibrosis was employed in multiple analyses. Other modalities included Computer Tomography (CT) and (Magnetic Resonance Imaging–Proton Density Fat Fraction) MRI-PDFF. Five studies identified cases using ICD codes.^21^^,^^24^^,^^34^^,^^37^^,^^39^ Liver biopsy which is the historical reference standard was used in three studies.^22^^,^^40^^,^^43^ This heterogeneity can introduce differential case-mix and misclassification. For example indices favor steatosis detection whereas ICD code cohorts may capture more clinically recognized disease, which reduces comparability across studies and may attenuate or inflate associations depending on the definition used.

Biological aging (BA) was assessed using established and novel metrics. Phenotypic Age (PhenoAge), calculated from clinical biochemistry markers, was the most frequently employed measure (*n* ​= ​10 studies^20^^,^^24^^,^^31^, ^32^, ^33^, ^34^^,^^36^, ^37^, ^38^^,^^42^^,^^44^). The Klemera-Doubal method (KDM-BA) was also highly prevalent (*n* ​= ​9 studies^20^^,^^24^^,^^32^, ^33^, ^34^, ^35^^,^^37^^,^^38^^,^^44^). Epigenetic clocks, which estimate age based on DNA methylation patterns, were used in four studies^22^^,^^23^^,^^40^^,^^43^; these included the Horvath, Hannum, DNAmPhenoAge, and DNAmGrimAge clocks. Leukocyte telomere length (LTL) was used as a cellular aging marker in three studies.^21^^,^^39^^,^^41^ Composite measures of physiological dysregulation, namely Homeostatic Dysregulation (HD) and Allostatic Load (AL), were used in three studies.^32^^,^^33^^,^^38^ One study developed a novel, machine-learning-based biological age metric.^33^

The role of biological age as a variable in the studies varied. In most studies, BA or BA acceleration was treated as the main exposure or predictor, with liver disease or related outcomes as the endpoints ^21^^,^^24^^,^^31^, ^32^, ^33^, ^34^^,^^36^, ^37^, ^38^, ^39^^,^^42^^,^^44^. In two studies, BA was the outcome, with liver disease status or fibrosis serving as the exposure.^20^^,^^23^ Five studies evaluated BA as a mediator, linking upstream exposures such as air pollution, metal mixtures, adiposity, or diet quality to liver outcomes 35, 36, 37^,^^42^^,^^44^. Several analyses also tested BA as a potential effect modifier, particularly in relation to genetic risk scores (PRS) or environmental toxicants.^24^^,^^34^^,^^37^^,^^40^^,^^44^

Outcomes spanned the full MASLD spectrum. Twelve studies focused on NAFLD/MASLD prevalence or incidence ^21^^,^^23^^,^^24^^,^^31^, ^32^, ^33^, ^34^, ^35^^,^^37^^,^^41^^,^^42^^,^^44^. Others evaluated significant or advanced fibrosis, cirrhosis, or elastography-based thresholds,^20^^,^^22^^,^^31^^,^^35^^,^^39^^,^^42^^,^^43^ biopsy-proven NASH,^22^^,^^34^^,^^40^ all-cause and cause-specific mortality,^33^^,^^38^^,^^39^ and related metabolic endpoints such as insulin resistance, prediabetes, and diabetes.^41^^,^^44^ Many studies examined multiple outcomes within the same analytic framework.

### 3.2 Association between biological aging and MASLD prevalence and incidence

Across multiple large cross-sectional datasets, accelerated BA was consistently associated with higher odds of MASLD/NAFLD. In NHANES 2005–2016, Liu et al. (2025) reported that each 1-SD increase in PhenoAge was associated with 75% higher odds of NAFLD defined by FLI ≥60 (OR 1.76, 95% CI 1.19–2.58), the strongest association among four aging measures examined; AL and HD showed more modest positive associations, whereas KDM-BA was weaker and not clearly significant.^32^ In pooled NHANES 1999–2018, Wu et al. (2025) found that individuals in the highest quartile of KDM-BA or PhenoAge had approximately three-fold higher odds of MASLD than those in the lowest quartile (KDM-BA Q4 vs Q1 OR 2.83, 95% CI 1.96–4.08; PhenoAge Q4 vs Q1 OR 2.90, 95% CI 1.88–4.46), after adjustment for sociodemographic and cardiometabolic factors.^36^ In the same study, a weighted mixture of urinary metals was associated with MASLD (WQS index OR 1.65, 95% CI 1.33–2.06).^36^

Not all BA metrics remained significant after adjustment. In a CT-based MASLD analysis from the MESA cohort, Gawrieh et al. (2025) observed that epigenetic age acceleration by the Hannum clock was associated with MASLD in minimally adjusted models, but this association attenuated after further adjustment for BMI, diabetes, and socioeconomic variables.^40^

Prospective cohort studies showed that BA also predicted incident NAFLD. In UK Biobank, Guo et al. (2025) followed 277,057 adults (2921 incident NAFLD and 2113 incident cirrhosis cases) and found graded associations between BA acceleration and incident disease.^34^ Compared with the lowest quartile, the highest quartile of KDMAge acceleration was associated with a 53% higher risk of incident NAFLD (HR 1.53, 95% CI 1.35–1.74) and similarly elevated risks for PhenoAge acceleration (HR 1.72, 95% CI 1.51–1.96); per-SD increases in BA acceleration were associated with about 17% higher risk for both NAFLD and cirrhosis.^34^

Using UK Biobank and the Dongfeng–Tongji cohort, Zhao and Wang et al. (2025) reported consistent findings across two large populations.^24^ In UKB, each 1-year increment in KDM-BA or PhenoAge acceleration was associated with a 6% higher hazard of incident NAFLD (KDM-BA HR 1.06, 95% CI 1.04–1.07; PhenoAge HR 1.06, 95% CI 1.05–1.06); “accelerated” versus “non-aging” BA corresponded to ∼35%–70% higher risk.^24^ Directionally similar but smaller effects were seen in the Dongfeng–Tongji cohort, where accelerated KDM-BA was associated with higher odds of ultrasound-defined NAFLD (OR 1.18, 95% CI 1.03–1.36; per-year KDM-BA OR 1.01, 95% CI 1.00–1.01).^24^ Extending these results, Zhang and Hai et al. (2025) found that BA acceleration (KDM-BA and PhenoAge) independently predicted incident NAFLD in UK Biobank, with per-SD HRs of 1.47 (KDM) and 1.38 (PhenoAge) and roughly two-to 2.5-fold higher risk in the highest versus lowest BA category.^37^

Leukocyte telomere length, used as a cellular aging marker, showed the expected inverse association with incident NAFLD. In UK Biobank, Tang et al. (2023) reported that longer LTL was modestly protective for NAFLD (HR 0.93 per IQR increase, 95% CI 0.89–0.96; HR 0.87, 95% CI 0.81–0.95 for highest vs lowest quartile), with similar inverse associations in an MRI-PDFF imaging subset and no clear effect for NASH.^21^ Mediation analysis suggested that telomere length mediated about 15% of the age–NAFLD association, and individuals with both short telomeres and high NAFLD polygenic risk had the highest disease risk.^21^

In a small prospective clinic cohort of patients with type 2 diabetes, Ping et al. (2017) found that greater leukocyte telomere shortening over 6 years was associated with higher odds of incident ultrasound-defined NAFLD (OR 1.5 per 1% shortening), independent of BMI, insulin, and blood pressure.^41^

### 3.3 Association of biological aging with MASLD severity and fibrosis

Several studies examined BA in relation to MASLD severity, particularly fibrosis. In a VCTE-based analysis of U.S. adults, Tong et al. (2024) reported that advanced fibrosis (but not MASLD itself) was associated with higher BA by blood-based clocks.^20^ Compared with individuals without advanced fibrosis, those with advanced fibrosis had, on average, 1.5 additional years of KDM-BA advance (β 1.50 years, 95% CI 0.23–2.77) and 1.0 additional year of PhenoAge advance (β 1.00 years, 95% CI 0.18–1.82) after adjustment.^20^

In NHANES 2017–2020, Zhao et al. (2025) used PhenoAge acceleration to predict VCTE-defined fibrosis among adults with MASLD.^31^ Each 1-year increase in PhenoAgeAccel was associated with higher odds of significant fibrosis (OR 1.01 per year), and individuals with accelerated aging (PhenoAgeAccel >0 vs ​≤ ​0) had 8% higher odds of fibrosis overall (OR 1.08, 95% CI 1.05–1.12).^31^ Associations were stronger in participants with obesity (OR 1.14, 95% CI 1.10–1.18) and in middle-aged adults (40–59 years: OR 1.14, 95% CI 1.07–1.22).^31^ Mendelian randomization in the same study suggested a potential causal role for cellular aging as genetically proxied longer telomere length was associated with lower odds of liver fibrosis/cirrhosis (OR 0.63, 95% CI 0.43–0.94), with no evidence of reverse causation.^31^

Biopsy-based data provided convergent evidence. In a single-center study of biopsy-proven NASH with F2–F3 fibrosis, Loomba et al. (2018) reported that epigenetic age acceleration (Horvath clock) was higher in NASH cases than in controls (median +2.8 years vs 0; *p* ​= ​0.03), with replication in external cohorts.^22^ Within NASH, stage F2 did not show clear EAA (*p* ​= ​0.12), whereas stage F3 showed modest elevation (*p* ​= ​0.04). EAA correlated with quantitative collagen content (ρ ​= ​0.45; *p* ​= ​0.004) and with the Enhanced Liver Fibrosis (ELF) panel (ρ ​= ​0.35; *p* ​= ​0.03).^22^

In a small liver tissue methylation study from Belgium, Van Dijck et al. (2025) analyzed 22 biopsies spanning the histologic MASLD spectrum (no MASLD, MASL, MASH F0–F4), with transcriptomic validation in 118 additional samples.^43^ Horvath EAA increased across MASLD stages and correlated with steatosis, ballooning, and fibrosis after FDR correction, whereas LiverClock EAA related primarily to stage and IEAA/HepClock EAA were not significant.^43^ They identified >22,000 CpG sites with stage-ordered methylation changes and >80,000 CpG sites associated with EAA, with substantial overlap between MASLD-stage– and EAA-linked loci, suggesting that epigenetic aging in liver tissue is tightly coupled to fibrotic progression.^43^

### 3.4 Biological aging and mortality among steatotic liver disease

Studies that assessed survival consistently linked higher BA to worse prognosis in steatotic liver disease. In NHANES III, Wang and Liu et al. (2024) followed 2199 adults with ultrasound-defined NAFLD for a median of 16 years (1077 deaths).^38^ Each 1-SD increase in BA was associated with higher all-cause mortality: KDM-BA HR 1.03 (95% CI 1.02–1.04), PhenoAge HR 1.07 (95% CI 1.06–1.08), and HD HR 1.39 (95% CI 1.29–1.50).^38^ Associations were directionally similar for cardiovascular and diabetes-related mortality and were often stronger in younger adults (<45 years).^38^

Kim et al. (2024) examined leukocyte telomere length and mortality among 3618 NHANES 1999–2002 participants with MASLD, followed for a median of 18.4 years (990 deaths).^39^ The shortest-LTL quartile had higher all-cause mortality than the longest (Q4 vs Q1 HR 1.41, 95% CI 1.03–1.94; *p* ​= ​0.035), with similar but less precise patterns for cardiovascular and cancer deaths.^39^ Gradients were most pronounced in adults ≥50 years, in men, and in non-Hispanic White participants.^39^

Using a novel ML-based BA metric (NC-BA), Deng et al. (2025) showed that higher BA acceleration predicted mortality among NAFLD patients.^33^ In NHANES NAFLD (*n* ​= ​1445; median follow-up 25 months; 26 deaths), participants with high versus low standardized age deviation (SAD) had markedly higher all-cause mortality (HR 6.25, 95% CI 1.44–27.05), with even larger HRs in men (HR 13.78, 95% CI 2.30–22.55), albeit with wide confidence intervals reflecting the small number of events.^33^

Zhao and Wang et al. (2025) modeled remaining life expectancy in UK Biobank and Dongfeng–Tongji, stratified by NAFLD and cirrhosis status and BA acceleration.^24^ From age 45, life expectancy was about 38.2 years (95% CI 37.6–38.9) in those without NAFLD compared with 35.4 years (34.6–36.2) in those with NAFLD, corresponding estimates were 38.2 years without cirrhosis versus 25.4 years with cirrhosis.^24^ Within NAFLD or cirrhosis strata, individuals without accelerated aging, particularly by PhenoAge, had longer life expectancy than those with BA acceleration.^24^

### 3.5 Genetic and environmental effect-modification

Several studies evaluated how genetic and environmental factors interact with BA in shaping NAFLD/MASLD risk. In UK Biobank, Zhao and Wang et al. (2025) reported strong joint effects of BA acceleration and NAFLD polygenic risk scores (PRS) on incident NAFLD.^24^ Using a joint reference group with low BA acceleration and low PRS, adjusted HRs rose to 1.85 (95% CI 1.63–2.10) for low BA/high PRS, 2.38 (95% CI 2.10–2.68) for high BA/low PRS, and 6.33 (95% CI 5.36–7.47) for high BA/high PRS.^24^

In another UK Biobank analysis, Guo et al. (2025) also found that BA acceleration and NAFLD PRS jointly influenced incident NAFLD and cirrhosis.^34^ Multiplicative interaction between PhenoAge acceleration and PRS reached statistical significance (*p*-interaction ​= ​0.02), with the association of BA acceleration appearing stronger in genetically lower-risk individuals, although the overall pattern still showed the highest risk among those with both elevated BA and high genetic risk.^34^

Single-locus genetic variation showed a similar pattern. In MESA, Gawrieh et al. (2025) reported that the PNPLA3 rs738409 ​G-allele amplified the association between Hannum-style epigenetic age and CT-defined MASLD.^40^ Among C-allele carriers, MASLD risk showed little gradient across epigenetic ages, whereas the positive association between epigenetic age acceleration and MASLD was stronger in G-allele carriers, consistent with effect modification by PNPLA3.^40^

Environmental co-exposures also appeared to potentiate aging-related risk. Zhang and Hai et al. (2025) found that long-term fine particulate matter (PM_2.5_) exposure and BA acceleration each independently predicted incident NAFLD in UK Biobank, with per-SD HR 1.07 (95% CI 1.04–1.10) for PM_2.5_ and HRs 1.38–1.47 for BA.^37^ Additive interaction modeling showed a modest supra-additive joint effect of high PM_2.5_ and high BA acceleration, with a relative excess risk due to interaction (RERI) of 0.13 (95% CI 0.00–0.27).^37^

Yang et al. (2025) examined urinary phthalates, BA, and glycemic outcomes among 3045 adults with MASLD in NHANES 1999–2018.^44^ Several phthalate metabolites for example MECPP, MEOHP, MiBP were associated with higher odds of insulin resistance and prediabetes, and a WQS mixture score was positively associated with insulin resistance, prediabetes, and diabetes.^44^ Both KDM-BA and PhenoAge were independently associated with higher odds of insulin resistance, prediabetes, and diabetes, and participants with both high phthalate exposure and high PhenoAge had the highest metabolic risk (diabetes OR ​= ​6 for high phthalates/high PA vs low/low), with significant interaction terms for insulin resistance, prediabetes, and diabetes (*p*-interaction <0.05).^44^

### 3.6 Mechanisms and pathways linking biological aging to MASLD spectrum

Beyond simple associations, several studies provided insight into mechanisms by which BA may contribute to MASLD onset and progression.

#### 3.6.1 The adiposity–aging–inflammation axis

Peng et al. (2025) analyzed NHANES 2017–2020 adults ≥50 years (*n* ​= ​3212) and identified a rapidly ascending BMI trajectory associated with higher odds of NAFLD (OR 2.21, 95% CI 1.29–3.77) and advanced fibrosis (OR 3.04, 95% CI 1.13–8.22) compared with a stable trajectory.^42^ Structural equation modeling showed that this effect operated in part through BA and inflammatory signaling. Higher PhenoAge predicted elevated hs-CRP, and this PhenoAge to hs-CRP pathway significantly mediated the associations with NAFLD and advanced fibrosis (indirect effects β ​= ​0.010 and 0.002, respectively).^42^

#### 3.6.2 Environmental exposures and aging-mediated toxicity

Wu et al. (2025) reported that urinary metal mixtures were associated with higher odds of MASLD (WQS OR 1.65, 95% CI 1.33–2.06) and that KDM-BA and PhenoAge mediated ∼20%–26% of this association.^36^ For specific metals such as cadmium and uranium, roughly half of the observed effect on MASLD risk appeared to be mediated through accelerated BA.^36^

#### 3.6.3 Lifestyle modification of aging-associated risk

Lifestyle factors, particularly diet, appear to modify the risk conveyed by accelerated biological aging. Zhang and Ding et al. (2025) examined KDM-BA, diet quality, and NAFLD in NHANES 2017–2018.^35^ Higher BA was associated with higher odds of NAFLD in a dose–response manner (KDM-BA Q4 vs Q1 OR 2.49, 95% CI 1.16–5.38; *p*-trend ​= ​0.018).^35^ A higher Healthy Eating Index (HEI-2015) score was associated with lower NAFLD odds (Q4 vs Q1 OR 0.64, 95% CI 0.46–0.87), and mediation analysis suggested that better diet quality modestly attenuated the BA–NAFLD association by about 6%.^35^

#### 3.6.4 Epigenetic and genetic evidence for fibrosis-linked aging signals

**Fig. 2:**
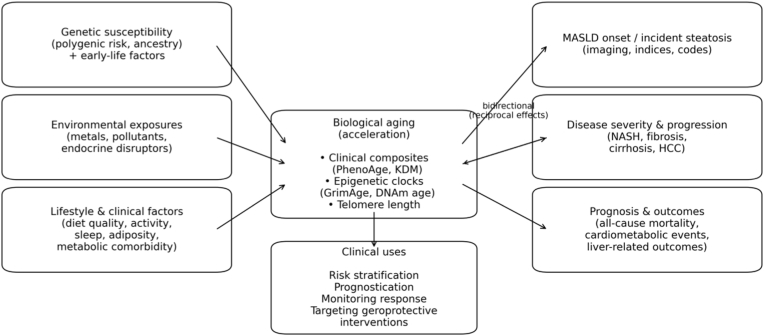
Conceptual framework Footnote: This conceptual framework illustrates the role of Biological Age (BA) as a central, modifiable risk integrator in the clinical management of Metabolic dysfunction–associated steatotic liver disease (MASLD) and MASLD-spectrum. BA is influenced by genetic susceptibility, environmental exposures, and lifestyle factors. It subsequently functions as both a mediator and effect modifier, influencing disease onset, progression, and mortality. BA can be deployed at key clinical decision points: for risk stratification to identify high-risk individuals prior to advanced disease, for prognostication to estimate fibrosis progression and mortality risk, and as a dynamic biomarker to monitor response to therapy and guide treatment intensity. The model posits that measuring BA provides a continuous readout of multisystem physiological decline, enabling a more proactive and personalized management paradigm for MASLD and MASLD related disorders. Abbreviations: NASH: Non-Alcoholic Steatohepatitis; HCC: hepatocellular carcinoma; KDM: Klemera-Doubal method.

Van Dijck et al. (2025) showed that liver specific epigenetic aging signals track closely with histologic MASLD progression, with thousands of CpG sites showing stage-ordered methylation changes and overlapping strongly with EAA-associated loci.^43^ In parallel, Zhao et al. (2025) used two-sample Mendelian randomization to show that genetically proxied longer telomere length was associated with lower risk of liver fibrosis/cirrhosis (OR 0.63, 95% CI 0.43–0.94), with no evidence of reverse causality.^31^ Together, these data support a model in which accelerated biological and cellular aging is not merely correlated with MASLD, but intertwined with the molecular pathways that drive fibrotic progression. Fig. 2 shows the conceptual framework that illustrates the role of BA as a central and modifiable risk integrator in management of MASLD and MASLD-spectrum disease.

## 4 Discussion

In this systematic review of 19 observational studies, higher BA, epigenetic age acceleration, and shorter telomere length were consistently associated with steatotic liver disease across the MASLD spectrum. Across diverse cohorts and measurement platforms, accelerated BA was linked to a higher prevalence of fatty liver/MASLD, greater hazards of incident NAFLD, more advanced fibrosis, and higher all-cause mortality among people with steatotic liver disease. Several studies also documented joint-exposure patterns in which BA combined with genetic or environmental risks to yield particularly high NAFLD risk, and mediation analyses suggested that BA accounted for a measurable proportion of the associations between environmental exposures and MASLD, with modest attenuation by healthier diet quality.

From a geroscience perspective, these findings are consistent with the view that the hallmarks of aging including genomic instability, epigenetic alterations, mitochondrial dysfunction, impaired proteostasis and autophagy, and chronic low-grade inflammation (“inflammaging”), are not passive correlates but active processes that drive MASLD onset and progression.^25^^,^^45^ Within this framework, MASLD can be viewed as a multisystem condition of systemic accelerated aging, with the liver acting as a sensitive indicator of cumulative physiological decline.^18^ BA measures remain associated with MASLD outcomes after adjustment for obesity and insulin resistance in many analyses, suggesting that BA captures a dimension of risk that is related to but not fully reducible to conventional metabolic factors.

Aging related processes remodel both hepatocytes and non-parenchymal cells through mechanisms such as senescence, dysregulated autophagy, and altered immune–stromal signaling 14, 15, 16, 17, 18. Senescent hepatocytes and non-parenchymal cells exhibit a senescence-associated secretory phenotype (SASP) characterized by p21 expression, DNA damage responses, and secretion of IL-6, IL-1β, TGF-β, matrix-remodeling enzymes, and Damage-Associated Molecular Patterns (DAMPs) that prime stellate cells, recruit macrophages, and maintain chronic injury loops 15, 16, 17, 18. This senescence–SASP axis provides a plausible mechanism that link upstream insults like adiposity and pollutants to fibrogenesis. It also aligns with the study findings in which BA partially mediated the impact of life-course adiposity on NAFLD and advanced fibrosis,^42^ mediated a substantial portion of the effect of metal mixtures on MASLD,^36^ and tracked with epigenetic aging signatures in liver tissue that correlate with steatosis, ballooning, and fibrosis.^43^

Multiple interrelated pathways further connect aging biology with MASLD spectrum development. Reduced NAD^+^ availability and sirtuin dysfunction, elevated mTOR activity with impaired autophagy, and defective mitophagy are all closely intertwined with key pathways underlying MASLD.^46^^,^^47^ Experimental work suggests that aberrant AMPK–mTOR signaling impairs autophagosome formation,^48^ while mitochondrial reactive oxygen species, mtDNA damage, and impaired β-oxidation promote lipid accumulation and hepatocellular stress.^49^ These abnormalities extend beyond hepatocytes as altered bile-acid signaling via FXR/TGR5^50^ and age-related shifts in the microbiome contribute to immune activation, endotoxemia, and metabolic dysregulation.^51^ Together these mechanisms support a model in which a biologically older liver is more vulnerable to metabolic disturbance.

Diet appears to be one of the few modifiers of BA. In NHANES, higher overall diet quality attenuated the association between KDM-BA and NAFLD after adjustment for confounders, corresponding to a modest but statistically significant reduction in disease risk.^35^ Randomized data support this biological plausibility. Long-term caloric restriction slows DNA-methylation-derived pace-of-aging measures,^52^ and Mediterranean-style dietary interventions have produced epigenetic rejuvenation over 12 months.^53^ These effects probably act through nutrient-sensing pathways such as AMPK–SIRT–mTOR, with downstream induction of autophagy and mitophagy and reductions in inflammatory markers including CRP and IL-6 in diet trials.^54^^,^^55^ In NAFLD/MASLD, Mediterranean-pattern diets improve hepatic steatosis and insulin resistance in randomized studies, suggesting that diet can alter disease biology rather than simply weight alone.^56^ Reflecting this, newer guidelines recommend Mediterranean-like dietary patterns to improve noninvasive and histologic markers of liver injury in MASLD.^57^ Diet is unlikely to fully reverse accelerated BA, but it is a pragmatic, scalable entry point that can be combined with weight loss and pharmacotherapy to modify the MASLD trajectory, even if only partially.

Our reviewed studies consistently situate BA in two complementary roles - both mediator and modifier. On the mediator side, NHANES mixture analyses showed that BA accounted for approximately 20–26% of the association between metal mixtures and MASLD, with mediation estimates approaching 50–60% for individual metals such as cadmium, cobalt, thallium, and uranium, supporting an exposome-aging-liver pathway.^36^ Peng et al. similarly identified PhenoAge and hs-CRP as sequential mediators linking rapidly ascending BMI trajectories to NAFLD and advanced fibrosis.^42^ As a modifier, BA amplified the impact of exogenous exposures. In UK Biobank, long-term PM_2.5_ exposure and BA acceleration had independent effects on incident NAFLD, with evidence of positive additive interaction.^37^ In NHANES adults with MASLD, phthalate mixtures and BA interacted to raise the odds of insulin resistance, prediabetes, and diabetes, with the highest risk in those with both high phthalate burdens and high PhenoAge.^44^ Environmental toxicants implicated in these cohorts are themselves linked to accelerated epigenetic aging and mitochondrial impairment 58, 59, 60, 61, 62, and a biologically older liver with established senescence and defective mitophagy is less capable of buffering additional hits.^14^^,^^15^^,^^47^ Together, these findings support BA as both a pathway through which exposures act and a factor that amplifies subsequent environmental or genetic insults.

Evidence for gene ​× ​BA interaction is emerging but mixed. In UK Biobank, Zhao et al. found the absolute risk of incident NAFLD highest among individuals with both high polygenic risk scores and accelerated BA, even though multiplicative interaction terms were not statistically significant, illustrating that clinically important risk concentration in joint-high groups can occur without formal multiplicative interaction.^24^ Guo et al. reported a statistically significant multiplicative interaction between PhenoAge acceleration and NAFLD-PRS (*p*-interaction ​= ​0.02), with the impact of BA appearing stronger in genetically lower-risk individuals, while absolute risk remained highest in those with both elevated BA and high PRS.^34^ At the single-locus level, PNPLA3 rs738409 magnified the association between Hannum-style epigenetic age and CT-defined MASLD in MESA; C-allele carriers showed minimal age gradients, whereas G-allele carriers had a steeper EAA–MASLD relationship.^40^ This hypothesis fits a model in which aging biology both integrates upstream exposures and conditions the liver's response to genetic risk.

At the molecular level, liver DNA methylation data directly link epigenetic aging to histologic severity. In the biopsy-based study by Van Dijck et al.,^43^ Horvath epigenetic age acceleration increased stepwise from no MASLD through MASL to MASH with advanced fibrosis and correlated with steatosis, ballooning, and fibrosis scores. They identified >20,000 CpG sites whose methylation changed in a stage-ordered fashion across the MASLD spectrum, with substantial overlap between CpGs associated with disease stage and those associated with epigenetic age acceleration. Pathway and motif analyses of these CpGs highlighted transcriptional programs involved in oxidative metabolism, redox balance, and inflammatory signaling. Importantly, NAFLD-related methylation changes appear at least partly reversible. In an independent bariatric-surgery cohort, NAFLD-associated CpG patterns partially reverted after substantial weight loss, with enrichment for Nuclear Respiratory Factor 1 (NRF1) and Estrogen-Related Receptor-α (ESRRA) binding motifs, transcription factors central to mitochondrial biogenesis and oxidative metabolism.^63^ Together, these findings support epigenetic aging in the liver as a fibrosis-linked signal that is dynamically modulated by intensive metabolic interventions.

Clock choice also matters. Obesity accelerates epigenetic aging in liver tissue, but the association can attenuate for blood-derived pan-tissue clocks once BMI is adjusted, whereas hepatocyte-enriched or liver-specific clocks retain strong associations with steatosis, ballooning, and fibrosis, underscoring the value of tissue-specific models.^19^^,^^64^ Nonetheless, peripheral blood methylation still provides clinically useful information. Loomba et al. reported that blood-based DNAm age acceleration correlated with morphometric hepatic collagen content in NASH, suggesting that peripheral epigenetic readouts can act as noninvasive proxies of liver fibrosis biology.^22^ Complementary Mendelian randomization findings indicate that genetically proxied longer telomere length—a marker of slower cellular aging—is associated with a lower risk of liver fibrosis/cirrhosis, supporting a role for aging pathways as upstream drivers of fibrogenesis rather than simply downstream consequences.^31^ However, the same genetic predisposition to longer telomeres is also linked to increased cancer risk,^65^ an important nuance for any therapeutic strategy targeting cellular aging.

Divergence across biological aging measures is expected because different metrics were trained on different endpoints and capture complementary biology. For near-term clinical risk stratification in MASLD, more practical clinical composite measures like PhenoAge or Klemera-Doubal method are often the most feasible because they rely on routinely available laboratory inputs and can be recalculated over time to monitor change. Epigenetic clocks like GrimAge or DNAm age acceleration may better reflect molecular aging and predict long-term outcomes, but they are more sensitive to batch effects, blood cell composition, and cost and availability constraints in routine care. Telomere length provides a biologically interpretable cellular aging signal, yet its associations can be weaker or more variable across platforms and populations. Accordingly, the most suitable index depends on the intended use-case. For example, screening and clinical implementation favor clinical composites while mechanistic inference and fibrosis biology may benefit from tissue-specific or liver-informed epigenetic clocks where available.

In terms of translating these insights to practice, the integration of BA metrics could refine existing noninvasive fibrosis pathways. Current algorithms progress from scores like FIB-4 to elastography and sometimes biopsy.^57^^,^^66^ Multisystem BA composites derived from routine labs (KDM-BA and PhenoAge) are inexpensive and reproducible. They could adjust the pre-test probability of advanced fibrosis, identifying midlife adults whose physiological risk outpaces their chronological age. For example, an elevated BA alongside a borderline FIB-4 might justify earlier elastography or specialist review.^8^^,^^20^^,^^67^ Furthermore, because BA is dynamic and improves with interventions like weight loss and cardiometabolic optimization, it holds promise as a treatment-response marker where serial biopsies are impractical.^22^^,^^52^^,^^63^^,^^68^^,^^69^ A declining BA under treatment could support continued noninvasive monitoring within a stepped-care framework.^20^^,^^57^^,^^66^

The therapeutic landscape for MASLD now centers on fibrosis endpoints. The 2024 FDA approval of resmetirom for noncirrhotic MASH with F2–F3 fibrosis underscores this shift.^4^^,^^5^^,^^70^ Resmetirom reduces hepatic fat and improves atherogenic lipids (LDL-C, ApoB, triglycerides).^4^^,^^5^ Since dyslipidemia and adverse lipidomic signatures are linked to faster epigenetic aging,^71^^,^^72^ resmetirom's cardiometabolic effects may intersect with pathways influencing BA acceleration. This connection suggests a hypothesis: patients with elevated BA might be enriched for therapeutic benefit. Incretin-based therapies provide a complementary mechanism. Tirzepatide leads to higher rates of MASH resolution and greater fibrosis improvement at 52 weeks than placebo,^73^ and semaglutide has demonstrated MASH resolution with evolving evidence on fibrosis.^74^^,^^75^ Mechanistically, GLP-1–based therapies activate AMPK/SIRT1 signaling, enhance autophagy, improve mitochondrial function, and dampen inflammatory pathways in liver models 76, 77, 78 which are processes central to aging biology. These overlaps justify using BA measures as exploratory endpoints to test whether patients with higher BA experience differential treatment effects and whether BA reductions track with histological improvements.

Preclinical data on senolytic strategies reveal both promise and complexity. While senescent stellate cells can restrain fibrosis in some mouse models,^79^ clearing senescent cells reduces age-related hepatic steatosis in others.^14^ In a medaka MASLD model, dasatinib plus quercetin reduced hepatic fat and fibrosis markers.^80^ Early human studies of this combination in idiopathic pulmonary fibrosis and diabetic kidney disease show feasibility and reduced senescence biomarkers, though they did not include hepatic outcomes.^81^^,^^82^ On the downside, indiscriminate senescent-cell clearance may impair regeneration in acute liver injury.^83^ Therefore, a precision approach is needed, targeting specific disease-driving senescent populations in the liver. Practical strategies could include curated SASP panels from proteomic resources, inclusion of GDF15 (which correlates with NAFLD severity), and, where feasible, tissue epigenetic age measures as mechanistic readouts 84, 85, 86.

BA is also associated with outcomes like mortality. In NAFLD/MASLD cohorts from NHANES, higher BA predicted all-cause and cause-specific mortality after adjustment,^33^^,^^38^^,^^39^ with the largest relative risks often seen in midlife adults.^38^ This aligns with the physiological processes captured by BA metrics. Clinical composites like PhenoAge are built from labs indexing inflammation, glycemia, and liver function,^8^ while methylation clocks such as GrimAge incorporate DNA-methylation surrogates for proteins like PAI-1 and GDF15, which are linked to cardiovascular and metabolic risk.^11^^,^^12^ Within MASLD populations, BA associates with advanced fibrosis on elastography^20^ and collagen burden on biopsy,^22^ tying it more closely to disease severity than to steatosis alone.

This synthesis has several important limitations:

First, study designs and phenotyping were heterogeneous. Steatotic liver disease was variously defined using biopsy, elastography (CAP/LSM), ultrasound, validated indices (e.g., FLI), or administrative codes, and fibrosis thresholds were not uniform. This heterogeneity can introduce misclassification and case-mix differences across studies. For example, studies that used ICD codes for disease ascertainment cohorts may represent more clinically recognized or advanced disease which can bias effect estimates in either direction and limits direct comparability. In many settings, nondifferential misclassification would be expected to attenuate associations toward the null, which contributed to our decision to use a narrative synthesis rather than a pooled meta-analysis.

Second, most included analyses were cross-sectional or relied on single time-point measurements of both biological aging and exposures, limiting temporal inference and increasing susceptibility to reverse causation. Mediation and interaction analyses rest on strong causal assumptions like no unmeasured confounding and correct model specification, and these are difficult to verify in observational data. Additionally, adjusting for potential mediators such as BMI or diabetes may introduce collider bias. Single measurements of diet, metals, and phthalate metabolites are vulnerable to within-person variability, and self-reported covariates add further measurement error.

Third, biological aging measurement itself was divergent. Epigenetic clocks, pace-of-aging measures, telomere length, and clinical composites capture overlapping but distinct constructs and were trained on different endpoints. Many studies used peripheral blood methylation to infer liver biology without cell-type deconvolution or liver-informed clocks, leaving residual bias from blood cell composition and batch effects. Clinical biological age measures may also be influenced by acute illness and medications, complicating interpretation in hospital-based or multimorbid cohorts. Generalizability is limited because much of the evidence comes from NHANES and the UK Biobank, where survey nonresponse and healthy-volunteer selection can constrain external validity, and some racial/ethnic and global regions are under-represented. Biological aging algorithms are often trained in specific populations and may not transport without recalibration across ancestry groups, socioeconomic contexts, or regions with different baseline inflammatory burdens and laboratory distributions. Because MASLD risk and access to diagnosis also vary by structural and social determinants, future work should evaluate model performance across diverse populations, report subgroup calibration and fairness metrics where feasible, and consider harmonized, context-specific reference ranges to avoid systematic over- or under-estimation of risk. Overlap across publications that reuse the same cohorts increases the risk of double counting and supported our narrative synthesis approach.

Fourth, statistical practice was not uniform. Not all NHANES analyses consistently accounted for survey weights, strata, and primary sampling units, and covariate selection was often driven by availability rather than explicit causal frameworks, increasing the risk of residual confounding. Few studies performed sensitivity analyses for unmeasured confounding, measurement error, or selection bias, and publication bias toward positive findings is possible. In addition, terminology and diagnostic criteria evolved over the review period (NAFLD, MAFLD, MASLD), and these constructs are not fully interchangeable as reclassification can change which individuals are considered cases and complicates comparisons over time.

Taken together, these limitations indicate that the current evidence base should be interpreted as providing preliminary etiologic signals rather than definitive causal evidence. Across cohorts, higher BA is consistently associated with greater fibrosis burden and adverse clinical outcomes and appears to lie on the pathway linking selected environmental exposures and genetic susceptibility to MASLD-related endpoints. However, strong causal inference, precise delineation of intermediate pathways, and any consideration of BA as a treatment-response biomarker will require prospective studies that standardize BA assays and calibration procedures, harmonize and validate MASLD definition and pre-specify mediation and interaction analyses within a causal framework, and include both liver outcomes and biomarkers targeting aging associated pathways like epigenetic age indices, senescence and fibrosis-related markers.

Overall certainty is low to moderate for causal inference because most data are observational, phenotyping and aging measures are heterogeneous, and residual confounding and selection bias remain plausible. In contrast, certainty is moderate for the direction of association, given the consistency of findings linking higher biological age with worse MASLD severity and outcomes across multiple cohorts and aging constructs.

## 5 Conclusion

In this systematic review, accelerated biological aging, assessed using clinical composite clocks, DNA methylation–based clocks, and telomere length, was consistently associated with the presence of NAFLD/MASLD, incident disease, fibrosis severity, and mortality among individuals with steatotic liver disease. Taken together, these findings support the idea that biological age captures a dimension of MASLD risk that is not explained by chronological age alone and likely reflects the accumulated impact of metabolic, environmental, and genetic factors on the liver. However, most available data are observational, frequently cross-sectional, and based on heterogeneous definitions of steatosis and fibrosis. Misclassification, residual confounding, and variation in BA algorithms and calibration limit the strength of inference, and the current evidence should be regarded as hypothesis-generating rather than definitive.

Moving from associative evidence to clinically useful application will require studies that are designed around biological aging. Prospective cohorts with standardized BA assays, harmonized MASLD definitions, and repeated measurements will be needed to assess temporality, within-person change, and incremental prognostic value beyond existing scores and elastography. Mediation and interaction analyses should be prespecified and powered to test hypotheses about how aging biology is associated with environmental, metabolic, and genetic factors to fibrosis and clinical outcomes. MASLD trials can incorporate validated BA measures as predefined exploratory endpoints to determine whether therapies that improve steatohepatitis and fibrosis also shift aging-related profiles. Only when these conditions are met will it be possible to determine whether targeting the pace of biological aging is a useful lever for altering the natural history of MASLD rather than a correlated marker of underlying risk.

## CRediT authorship contribution statement

**Chukwuemeka E. Ogbu:** Writing – review & editing, Writing – original draft, Validation, Methodology, Data curation, Conceptualization, Investigation, Visualization. **Stella C. Ogbu:** Writing – review & editing, Writing – original draft, Validation, Visualization. **Chidera P. Ogbu:** Writing – review & editing, Writing – original draft, Data curation. **Chinazor Umerah:** Writing – review & editing, Validation, Supervision, Resources.

## Informed consent

Not applicable.

## Ethics statement

Ethical review was obtained from Cape Fear Valley Health. The study was reviewed and deemed exempt from Institutional Review Board (IRB) oversight. Therefore, no ethical approval code was issued and no clinical trial registration is applicable.

## Clinical trial registration

Not applicable. This work is a systematic review of observational studies and did not involve the conduct of a clinical trial.

## Declaration of generative AI and AI-assisted technologies in the writing process

No generative AI tools were used in the conception, drafting, editing, or formatting of this manuscript. All text and analyses were produced directly by the authors.

## Funding

This research received no external funding.

## Data availability

No new primary data were generated for this study. All data synthesized in this systematic review were obtained from previously published articles cited in the reference list.

## Conflict of interest

The authors declare that there are no conflicts of interest related to this work.

## References

[1] Rinella M.E., Lazarus J.V., Ratziu V. (2023). A multisociety Delphi consensus statement on new fatty liver disease nomenclature. Hepatology.

[2] Younossi Z.M., Kalligeros M., Henry L. (2025). Epidemiology of metabolic dysfunction-associated steatotic liver disease. Clin Mol Hepatol.

[3] Le P., Tatar M., Dasarathy S. (2025). Estimated burden of metabolic dysfunction-associated steatotic liver disease in US adults, 2020 to 2050. JAMA Netw Open.

[4] Harrison S.A., Taub R., Neff G.W. (2023). Resmetirom for nonalcoholic fatty liver disease: a randomized, double-blind, placebo-controlled phase 3 trial. Nat Med.

[5] Harrison S.A., Bedossa P., Guy C.D. (2024). A phase 3, randomized, controlled trial of resmetirom in NASH with liver fibrosis. N Engl J Med.

[6] Bland J.S. (2018). Age as a modifiable risk factor for chronic disease. Integr Med.

[7] Klemera P., Doubal S. (2006). A new approach to the concept and computation of biological age. Mech Ageing Dev.

[8] Liu Z., Kuo P.L., Horvath S. (2018). A new aging measure captures morbidity and mortality risk across diverse subpopulations from NHANES IV: a cohort study. PLoS Med.

[9] Levine M.E., Lu A.T., Quach A. (2018). An epigenetic biomarker of aging for lifespan and healthspan. Aging.

[10] Horvath S. (2013). DNA methylation age of human tissues and cell types. Genome Biol.

[11] Lu A.T., Quach A., Wilson J.G. (2019). DNA methylation GrimAge strongly predicts lifespan and healthspan. Aging.

[12] Belsky D.W., Caspi A., Corcoran D.L. (2022). DunedinPACE, a DNA methylation biomarker of the pace of aging. eLife.

[13] Huang X., Huang L., Lu J. (2025). The relationship between telomere length and aging-related diseases. Clin Exp Med.

[14] Ogrodnik M., Miwa S., Tchkonia T. (2017). Cellular senescence drives age-dependent hepatic steatosis. Nat Commun.

[15] Tchkonia T., Zhu Y., van Deursen J. (2013). Cellular senescence and the senescent secretory phenotype: therapeutic opportunities. J Clin Investig.

[16] Huang W., Hickson L.J., Eirin A. (2022). Cellular senescence: the good, the bad and the unknown. Nat Rev Nephrol.

[17] Prašnikar E., Borišek J., Perdih A. (2021). Senescent cells as promising targets to tackle age-related diseases. Ageing Res Rev.

[18] Papatheodoridi A.M., Chrysavgis L., Koutsilieris M. (2020). The role of senescence in the development of nonalcoholic fatty liver disease and progression to nonalcoholic steatohepatitis. Hepatology.

[19] Horvath S., Erhart W., Brosch M. (2014). Obesity accelerates epigenetic aging of human liver. Proc Natl Acad Sci USA.

[20] Tong C., Xue Y., Wang W. (2024). Advanced liver fibrosis, but not MASLD, is associated with accelerated biological aging: a population-based study. BMC Public Health.

[21] Tang L., Li D., Ma Y. (2023). The association between telomere length and non-alcoholic fatty liver disease: a prospective study. BMC Med.

[22] Loomba R., Gindin Y., Jiang Z. (2018). DNA methylation signatures reflect aging in patients with nonalcoholic steatohepatitis. JCI Insight.

[23] Xia M., Li W., Lin H. (2024). DNA methylation age acceleration contributes to the development and prediction of non-alcoholic fatty liver disease. GeroScience.

[24] Zhao Y., Wang Y., Chen L. (2025). Accelerated biological aging, genetic susceptibility, and non-alcoholic fatty liver disease: two prospective cohort studies. Nutrients.

[25] Li Y., Adeniji N.T., Fan W. (2022). Non-alcoholic fatty liver disease and liver fibrosis during aging. Aging Dis.

[26] Belsky D.W., Moffitt T.E., Cohen A.A. (2018). Eleven telomere, epigenetic clock, and biomarker-composite quantifications of biological aging: do they measure the same thing?. Am J Epidemiol.

[27] Page M.J., McKenzie J.E., Bossuyt P.M. (2021). The PRISMA 2020 statement: an updated guideline for reporting systematic reviews. BMJ.

[28] Wells G.A., Shea B., O'Connell D. (2011). https://www.ohri.ca/programs/clinical_epidemiology/oxford.asp.

[29] Moola S., Munn Z., Sears K. (2015). Conducting systematic reviews of association (etiology): the *Joanna* Briggs Institute's approach. Int J Evid Base Healthc.

[30] Campbell M., McKenzie J.E., Sowden A. (2020). Synthesis without meta-analysis (SWiM) in systematic reviews: reporting guideline. Br Med J.

[31] Zhao J., Zhou H., Wu R. (2025). Biological aging accelerates hepatic fibrosis: insights from the NHANES 2017–2020 and genome-wide association study analysis. Ann Hepatol.

[32] Liu G., Mao Q., Tian X. (2025). Association of biological aging and the prevalence of nonalcoholic fatty liver disease: a population-based study. BMC Gastroenterol.

[33] Deng L., Huang J., Yuan H. (2025). Biological age prediction and NAFLD risk assessment: a machine learning model based on a multicenter population in Nanchang, Jiangxi, China. BMC Gastroenterol.

[34] Guo R., Hu J., Yang H. (2024). Associations of accelerated biological ageing, genetic predisposition, and life expectancy with non-alcoholic fatty liver disease and cirrhosis: a prospective cohort study [preprint]. SSRN.

[35] Zhang X., Ding Z., Yan Y. (2025). The effect of healthy eating index-2015 in the associations of biological aging and non-alcoholic fatty liver disease: an interaction and mediation analysis. J Health Popul Nutr.

[36] Wu Y.P., Feng J., Zhang Y.Y. (2025). Biological ageing mediates the associations between urinary metals and metabolic dysfunction-associated steatotic liver disease. Ecotoxicol Environ Saf.

[37] Zhang X., Hai P., Xue J. (2025). Combined effect of biological age and fine particulate matter pollution with risk of non-alcoholic fatty liver disease in the UK biobank: a prospective cohort study. Am J Epidemiol.

[38] Wang H., Liu Z., Fan H. (2024). Association between biological aging and the risk of mortality in individuals with non-alcoholic fatty liver disease: a prospective cohort study. Arch Gerontol Geriatr.

[39] Kim D., Danpanichkul P., Wijarnpreecha K. (2024). Leukocyte telomere shortening in metabolic dysfunction-associated steatotic liver disease and all-cause/cause-specific mortality. Clin Mol Hepatol.

[40] Gawrieh S., Yao J., Guo X. (2025). Biological age, PNPLA3, and risk of metabolic dysfunction-associated steatotic liver disease. Clin Gastroenterol Hepatol.

[41] Ping F., Li Z.Y., Lv K. (2017). Deoxyribonucleic acid telomere length shortening can predict the incidence of non-alcoholic fatty liver disease in patients with type 2 diabetes mellitus. J Diabetes Investig.

[42] Peng H., Zhao Z., Gong J. (2025). BMI trajectories are associated with NAFLD and advanced fibrosis *via* aging-inflammation mediation. BMC Public Health.

[43] Van Dijck E., Van Laere S., Logie E. (2025). Gradual DNA methylation changes reveal transcription factors implicated in metabolic dysfunction-associated steatotic liver disease progression and epigenetic age acceleration. Clin Epigenet.

[44] Yang Y., Wan S., Yu L. (2025). Phthalates exposure, biological aging, and increased risks of insulin resistance, prediabetes, and diabetes in adults with metabolic dysfunction-associated steatotic liver disease. Diabetes Metab.

[45] He Y., Su Y., Duan C. (2023). Emerging role of aging in the progression of NAFLD to HCC. Ageing Res Rev.

[46] Zeng C., Chen M. (2022). Progress in nonalcoholic fatty liver disease: SIRT family regulates mitochondrial biogenesis. Biomolecules.

[47] Undamatla R., Fagunloye O.G., Chen J. (2023). Reduced mitophagy is an early feature of NAFLD and liver-specific PARKIN knockout hastens the onset of steatosis, inflammation and fibrosis. Sci Rep.

[48] Marcondes-de-Castro I.A., Reis-Barbosa P.H., Marinho T.S. (2023). AMPK/mTOR pathway significance in healthy liver and non-alcoholic fatty liver disease and its progression. J Gastroenterol Hepatol.

[49] Raza S., Rajak S., Yen P.M. (2024). Autophagy and hepatic lipid metabolism: mechanistic insight and therapeutic potential for MASLD. NPJ Metab Health Dis.

[50] Chiang J.Y.L., Ferrell J.M. (2020). Bile acid receptors FXR and TGR5 signaling in fatty liver diseases and therapy. Am J Physiol Gastrointest Liver Physiol.

[51] An L., Wirth U., Koch D. (2022). The role of gut-derived lipopolysaccharides and the intestinal barrier in fatty liver diseases. J Gastrointest Surg.

[52] Waziry R., Ryan C.P., Corcoran D.L. (2023). Author Correction: effect of long-term caloric restriction on DNA methylation measures of biological aging in healthy adults from the CALERIE trial. Nat Aging.

[53] Gensous N., Garagnani P., Santoro A. (2020). One-year Mediterranean diet promotes epigenetic rejuvenation with country- and sex-specific effects: a pilot study from the NU-AGE project. GeroScience.

[54] de Cabo R., Mattson M.P. (2019). Effects of intermittent fasting on health, aging, and disease. N Engl J Med.

[55] Casas R., Sacanella E., Urpí-Sardà M. (2014). The effects of the Mediterranean diet on biomarkers of vascular wall inflammation and plaque vulnerability in subjects with high risk for cardiovascular disease. A randomized trial. PLoS One.

[56] George E.S., Reddy A., Nicoll A.J. (2022). Impact of a Mediterranean diet on hepatic and metabolic outcomes in non-alcoholic fatty liver disease: the *MEDINA* randomised controlled trial. Liver Int.

[57] Tacke F., Horn P., Wai-Sun Wong V. (2024). EASL–EASD–EASO Clinical Practice guidelines on the management of metabolic dysfunction-associated steatotic liver disease (MASLD). J Hepatol.

[58] Mi P., Cao X., Feng H. (2025). Association of blood cadmium levels with epigenetic age acceleration in U.S. adults aged > 50 years. Front Public Health.

[59] Ward-Caviness C.K., Nwanaji-Enwerem J.C., Wolf K. (2016). Long-term exposure to air pollution is associated with biological aging. Oncotarget.

[60] Gao X., Huang J., Cardenas A. (2022). Short-term exposure of PM_2.5_ and epigenetic aging: a quasi-experimental study. Environ Sci Technol.

[61] Zheng Z., Xu X., Zhang X. (2013). Exposure to ambient particulate matter induces a NASH-like phenotype and impairs hepatic glucose metabolism in an animal model. J Hepatol.

[62] Moore M.P., Cunningham R.P., Meers G.M. (2022). Compromised hepatic mitochondrial fatty acid oxidation and reduced markers of mitochondrial turnover in human NAFLD. Hepatology.

[63] Ahrens M., Ammerpohl O., von Schönfels W. (2013). DNA methylation analysis in nonalcoholic fatty liver disease suggests distinct disease-specific and remodeling signatures after bariatric surgery. Cell Metab.

[64] Tong H., Guo X., Jacques M. (2024). Cell-type specific epigenetic clocks to quantify biological age at cell-type resolution. Aging.

[65] Haycock P.C., Burgess S., Nounu A. (2017). Association between telomere length and risk of cancer and non-neoplastic diseases: a Mendelian randomization study. JAMA Oncol.

[66] Rinella M.E., Neuschwander-Tetri B.A., Siddiqui M.S. (2023). AASLD Practice guidance on the clinical assessment and management of nonalcoholic fatty liver disease. Hepatology.

[67] Zhang R., Wu M., Zhang W. (2023). Association betweenlife's essential 8 andbiological ageing amongUS adults. J Transl Med.

[68] Fraszczyk E., Luijten M., Spijkerman A.M.W. (2020). The effects of bariatric surgery on clinical profile, DNA methylation, and ageing in severely Obese patients. Clin Epigenet.

[69] Fiorito G., Caini S., Palli D. (2021). DNA methylation-based biomarkers of aging were slowed down in a two-year diet and physical activity intervention trial: the *DAMA* study. Aging Cell.

[70] U.S. Food and Drug Administration (March 14, 2024). FDA approves first treatment for patients with liver scarring due to fatty liver disease (Rezdiffra [resmetirom]). News release.

[71] Liu D., Ahmad Aziz N., Landstra E.N. (2023). The lipidomic correlates of epigenetic aging across the adult lifespan: a population-based study. Aging Cell.

[72] Lin L., Kiryakos J., Ammous F. (2024). Epigenetic age acceleration is associated with blood lipid levels in a multi-ancestry sample of older U.S. adults. BMC Med Genom.

[73] Loomba R., Hartman M.L., Lawitz E.J. (2024). Tirzepatide for metabolic dysfunction-associated steatohepatitis with liver fibrosis. N Engl J Med.

[74] Newsome P.N., Buchholtz K., Cusi K. (2021). A placebo-controlled trial of subcutaneous semaglutide in nonalcoholic steatohepatitis. N Engl J Med.

[75] Sanyal A.J., Newsome P.N., Kliers I. (2025). Phase 3 trial of semaglutide in metabolic dysfunction-associated steatohepatitis. N Engl J Med.

[76] He Q., Sha S., Sun L. (2016). GLP-1 analogue improves hepatic lipid accumulation by inducing autophagy *via* AMPK/mTOR pathway. Biochem Biophys Res Commun.

[77] Papakonstantinou I., Tsioufis K., Katsi V. (2024). Spotlight on the mechanism of action of semaglutide. Curr Issues Mol Biol.

[78] Jara M., Norlin J., Kjær M.S. (2025). Modulation of metabolic, inflammatory and fibrotic pathways by semaglutide in metabolic dysfunction-associated steatohepatitis. Nat Med.

[79] Krizhanovsky V., Yon M., Dickins R.A. (2008). Senescence of activated stellate cells limits liver fibrosis. Cell.

[80] Yakubo S., Abe H., Li Y. (2024). Dasatinib and quercetin as senolytic drugs improve fat deposition and exhibit antifibrotic effects in the medaka metabolic dysfunction-associated steatotic liver disease model. Diseases.

[81] Justice J.N., Nambiar A.M., Tchkonia T. (2019). Senolytics in idiopathic pulmonary fibrosis: results from a first-in-human, open-label, pilot study. EBioMedicine.

[82] Hickson L.J., Langhi Prata L.G.P., Bobart S.A. (2019). Senolytics decrease senescent cells in humans: preliminary report from a clinical trial of Dasatinib plus Quercetin in individuals with diabetic kidney disease. EBioMedicine.

[83] Ma X., Huang T., Chen X. (2025). Molecular mechanisms in liver repair and regeneration: from physiology to therapeutics. Signal Transduct Targeted Ther.

[84] Basisty N., Kale A., Jeon O.H. (2020). A proteomic atlas of senescence-associated secretomes for aging biomarker development. PLoS Biol.

[85] Bilson J., Scorletti E., Bindels L.B. (2021). Growth differentiation factor-15 and the association between type 2 diabetes and liver fibrosis in NAFLD. Nutr Diabetes.

[86] Sanfeliu-Redondo D., Gibert-Ramos A., Gracia-Sancho J. (2024). Cell senescence in liver diseases: pathological mechanism and theranostic opportunity. Nat Rev Gastroenterol Hepatol.

